# Supplementary material for: A Critical View of the Application of the APEX Software (Aqueous Photochemistry of Environmentally-Occurring Xenobiotics) to Predict Photoreaction Kinetics in Surface Freshwaters
Source: Molecules. 2019 Dec 18;25(1):9. doi: 10.3390/molecules25010009 (PMC7017383; doi:10.3390/molecules25010009)
Supplement: Supplementary file 1 [file molecules-25-00009-s001.zip › 3.2.4_gcc-4.4.0.zip!/3.2.4_gcc-4.4.0/3.2.4_gcc-4.4.0/doc/octave/html/interpreter/Surface-Properties.html]

Surface Properties - Untitled


Next: Searching Properties,
Previous: Patch Properties,
Up: Graphics Object Properties


---

##### 15.2.2.8 Surface Properties

`xdata` `ydata` `zdata`: The data determining the surface. The `xdata` and `ydata` elements are vectors and `zdata` must be a matrix. `keylabel`: The text of the legend entry corresponding to this surface. Note that this property is not compatible with matlab and may be removed in a future version of Octave.
